# Developing normative values and predictive models for the 6‐minute walk test across diverse adolescent developmental stages

**DOI:** 10.1002/ejsc.12169

**Published:** 2024-07-29

**Authors:** Hatem Ghouili, Ismail Dergaa, Amel Dridi, Zouhaier Farhani, Nejmeddine Ouerghi, Mohamed Ben Aissa, Nadhir Hammami, Anissa Bouassida, Noomen Guelmami, Nizar Souissi, Katja Weiss, Thomas Rosemann, Lamia Ben Ezzeddine, Beat Knechtle

**Affiliations:** ^1^ Research Unit: Sports Science Health and Movement High Institute of Sport and Physical Education of Kef University of Jendouba Kef Tunisia; ^2^ Primary Health Care Corporation (PHCC) Doha Qatar; ^3^ Research Unit: Physical Activity Sport, and Health UR18JS01 National Observatory of Sport Tunis Tunisia; ^4^ High Institute of Sport and Physical Education of Sfax University of Sfax Sfax Tunisia; ^5^ High Institute of Sport and Physical Education of Gafsa University of Gafsa Gafsa Tunisia; ^6^ High Institute of Sport and Physical Education Ksar‐Saïd University of Manouba Mannouba Tunisia; ^7^ Department of Health Sciences (DISSAL) Postgraduate School of Public Health University of Genoa Genoa Italy; ^8^ Institute of Primary Care University of Zurich Zurich Switzerland; ^9^ Medbase St. Gallen Am Vadianplatz St. Gallen Switzerland; ^10^ Research Laboratory: Sports Performance Optimisation Research LR09SEP01 National Centre for Sports Medicine and Science Tunis Tunisia

**Keywords:** 6MWD, children, functional capacity, healthy, percentile curves, prediction equation, puberty, youth

## Abstract

The six‐minute walking test (6MWT) is commonly used to measure functional capacity in field settings, primarily through the distance covered. This study aims to establish reference curves for the six‐minute walking distance (6MWD) and peak heart rate (PHR) and develop a predictive equation for cardiovascular capacity in Tunisian children and adolescents. A total of 1501 participants (706 boys and 795 girls), aged 10–18 years, were recruited from schools in Tunisia. The Lambda (L), Mu (M), and Sigma (S) methods (LMS method) were employed to develop smoothed percentile curves for 6MWD and PHR. Multivariate linear regression was utilized to formulate a prediction equation for 6MWD. Smoothed percentiles (3rd, 10th, 25th, 50th, 75th, 90th, and 97th) for 6MWD and PHR were presented with age. All variables showed a strong positive correlation (*p* < 0.001) with a six‐minute walking distance (*r* ranged from 0.227 to 0.558 for girls and from 0.309 to 0.610 for boys), except resting heart rate, which showed a strong negative correlation (girls: *r* = −0.136; boys: *r* = −0.201; *p* < 0.001). Additionally, PHR showed a weak correlation (*p* > 0.05). The prediction equations, based on age as the primary variable, were established for both genders. For boys: 6MWD = 66.181 + 38.142 × Age (years) (*R* ^2^ = 0.372; Standard Error of Estimate (SEE) = 122.13), and for girls: 6MWD = 105.535 + 28.390 × Age (years) (*R* ^2^ = 0.312; SEE = 103.66). The study provides normative values and predictive equations for 6MWD and PHR in Tunisian children and adolescents. These findings offer essential tools for identifying, monitoring, and interpreting cardiovascular functional deficits in clinical and research settings.

## Highlights

- Established reference curves for six‐minute walking distance (6MWD) and peak heart rate (PHR) from the data of 1501 children and adolescents aged 10–18 years were recruited from multicenter schools in Tunisia.
- Developed predictive equations for 6MWD based on age using multivariate linear regression.
- Provides normative values and predictive equations for 6MWD and PHR, offering valuable tools for identifying and monitoring cardiovascular functional deficits in clinical and research settings.

## 1 INTRODUCTION

Functional capacity includes a person's ability to perform everyday activities such as walking, lifting, pushing, pulling, or manipulating objects (Fore et al., 2015; Guralnik et al., 1995). The gold standard for objective assessment of this capacity is the cardiopulmonary exercise test (CPET), a sophisticated laboratory test that requires expensive specialized equipment and a team of professionals, and access to CPET remains limited, especially in primary care (ATS, 2002; Singh et al., 2014).

Currently, the most widely used valid and reliable field measure of functional capacity is the six‐minute walk test (6MWT) that assesses distance covered. The test is typically performed at a self‐selected pace on a hard surface such as a hospital corridor (Andrianopoulos et al., 2015; ATS, 2002; Singh et al., 2014). The 6MWT is valued for its speed, simplicity, cost‐effectiveness, ease of administration, and ability to better reflect daily activities compared to other exercises (Enright, 2003; Salvi et al., 2020). The maximum oxygen consumption (VO_2_max), as determined during a CPET, primarily evaluates physiological health aspects, whereas the 6MWD, measured during the 6MWT, more directly assesses limitations in physical activity (Bui et al., 2017). Therefore, the 6MWT complements CPET in assessing the functional status of patients and may provide a viable alternative when CPET is not available (Rostagno & Gensini, 2008).

The utility of 6MWT extends to the assessment of exercise tolerance in various pathological conditions, including cardiovascular disease, asthma, pulmonary arterial hypertension, neuromuscular disease, and arthritis (Bartels et al., 2013; Demir & Küçükoğlu, 2015; Ferreira et al., 2022; Pereira et al., 2015; Witherspoon et al., 2019). In recent decades, numerous studies have been performed in both adults and healthy children to establish reference curves for 6MWD (Goemans et al., 2013; Kasović et al., 2021; Ulrich et al., 2013; Vandoni et al., 2018). In clinical practice, these reference curves play a central role as screening tools in the crucial stages of childhood and help in identifying individuals with abnormal performance values (Borghi et al., 2006). To gain a deeper insight into the factors influencing 6MWD as a dependent variable, several independent variables such as age, height, waist circumference (WC), and body fat percentage were analyzed using regression analysis (Almeida et al., 2019; Geiger et al., 2007; Mylius et al., 2016).

Nevertheless, it is important to recognize that there are significant differences in 6MWT outcomes between different countries (Cacau et al., 2016; Casanova et al., 2011). These differences can be substantial, with variations of up to 159 m observed among children of diverse nationalities (Vandoni et al., 2018). Factors such as demographic characteristics (Almeida et al., 2019), ethnicity (Ben Saad, Prefaut, Tabka, et al., 2009b), dietary habits (D'Silva et al., 2012; Shepherd et al., 2015), and level of physical activity (Breda et al., 2013) may contribute to these differences in performance.

In Tunisia, there has been significant progress in establishing reference curves for various health indicators. These include growth metrics, body mass index (BMI), WC among healthy children and adolescents, as well as reference curves for heart rate and VO_2_max specifically tailored for young soccer players (Ghouili et al., 2018, 2020, 2021, 2023). However, regarding the 6MWT, the only existing prediction equation was developed from a 2009 study, which was based on data from 200 participants, comprising an equal number of girls and boys, all between the ages of six and 16 years (Ben Saad, Prefaut, Missaoui, et al., 2009a).

Based on the observed variations in 6MWT outcomes internationally and the limited scope of existing Tunisian data on pediatric functional capacity, the aim of the present study was to establish comprehensive reference curves for the 6MWT and peak heart rate (PHR) and to develop prediction models specifically for measurements of Tunisian children aged 10–18 years. Moreover, we seek to compare between our reference curves developed and those from other countries as well as between our model estimated and the Tunisian model 2009.

## 2 METHODS

This study began on November 2022 and continued until the end of May 2023 in educational establishments under the supervision of the research unit of the Higher Institute of Sport and Physical Education of Kef (ISSEP), Kef, Tunisia.

### 2.1 Study population

The selection of a sample of children aged 10–18 years was established using the stratified random analysis technique. We first divided Tunisia into three large zones (North, Center, and South). Then, we randomly selected three governorates from a total of 24 governorates (one governorate per zone).

We contacted their Regional Education Commissions. After our study was approved, all schools that belong to these Regional Education Commissions and met these two conditions: (1) they contain the 5th and 6th year levels for primary schools and all levels of study for preparatory schools and high schools; (2) the average number of students per classroom exceeding 25 were included in the final draw list. In the final stage, we randomly selected two primary schools, two preparatory schools, and one secondary school per each governorate. After signing the informed consent, parents or guardians reported their child's health status, whether they had common chronic illnesses such as asthma, diabetes, cardiovascular disease, and epilepsy. All children with any of these reported chronic illnesses were excluded from study participation. Subjects and their parents/guardians were also informed that their participation was voluntary and that they could withdraw from the study at any time without negative consequences.

### 2.2 Anthropometric measurements

Anthropometric measurements were performed by six examiners well trained in standard anthropometric techniques (WHO, 1995). The child must be barefoot or in thin socks and wear light clothing.

For the Height, the subject should stand on a flat surface with weight distributed evenly on both feet, heels together, and the head positioned so that the line of vision is perpendicular to the body. The arms hang freely at the sides and the head, back, buttocks, and heels are in contact with the vertical board of the stadiometer. Height was measured using a Seca 206 portable stadiometer (Hamburg, Germany) to the nearest 0.1.

For Body mass measurement, the child is placed motionless on the scale so that the weight is distributed equally around the center of the scale. Body mass was measured to the nearest 0.2 kg using a Tanita BF‐681 W electronic scale (Tokyo, Japan).

In sitting height (SH), subjects who were seated on a table placed in front of the stadiometer, its dimensions constructed using the recommendations of Cameron (1982). The subject places his back, buttocks, and occipital muscles in contact with the vertical plane of the wall. The knees form right angles (90°), the hands on the thighs and the head positioned in the Frankfurt plane. Leg length (LL) was calculated for each subject as height (cm) minus SH (cm).

The WC measurement site was located midway between the lowest rib and the top of the iliac crest. We asked subjects to stand with their weight distributed equally on both legs, keep their abdomen relaxed, and breathe gently at the time of measurement. WC was estimated using a flexible non‐elastic tape to the nearest centimeter.

### 2.3 6MWT protocol

The 6MWT was performed in accordance with American Thoracic Society (ATS) recommendations (ATS, 2002). Participants were asked to walk as fast as possible at a self‐selected pace along a flat and straight corridor measuring 30‐m in length for six minutes after a 10‐min rest period. They were allowed to stop whenever they wanted. Evaluators encouraged participants with standardized phrases as described by the ATS. To ensure that the children understood the instructions, a trial test was conducted on a portion of the course. The children were divided into small groups to complete the test. The final score was expressed in distance covered (m) in a 6‐min period. Heart rate (bpm) was measured using the Polar Team Sport system (Polar‐Electro OY, Kempele, Finland) after a period of rest in a seated position for at least 5 min. Recording continued until the end of the test.

All the testing sessions were conducted at the same time of the day (8–9 a.m.) to minimize the effects of diurnal variation in the measured parameters (Dergaa et al., 2019, 2020, 2021).

### 2.4 Statistical analysis

Descriptive statistics were presented as mean and standard deviation. Kolmogorov–Smirnov test was used to check normal distribution of data. Differences between genders and between consecutive age groups were calculated using one‐way analysis of variance with a post hoc comparison test between pairs. To calculate the correlations between all the independent variables and 6MWD, we used the Pearson correlation coefficient (*r*)

#### 2.4.1 Prediction of regression models

The stepwise multivariate linear regression method was used to identify the predictors (sex, age, height, weight, SH, WC, and resting heart rate (RHR)) of 6MWD after checking the regression assumptions (homoscedasticity, multicollinearity, and normal distribution residuals). The standardized beta correlation coefficient (*β*) and the coefficient of determination *R* ^2^ were used to assess the quality of fit of the model. Examination of the accuracy and variability of the forecasting equations was carried out by analysis of the Bland, Altman graphical method (Bland & Altman, 1999).

#### 2.4.2 Smoothing curves

In this study, we analyzed each set of data using the LMS method (Cole & Green, 1992). This method makes it possible to obtain smoothed percentile curves (P3, P10, P25, P50, P75, P90, and P97) based on three curves called L (lambda), M (mu), and S (sigma). The M and S curves correspond to the median and the coefficient of variation at each age, while the L curve expresses the power necessary to transform the data into a normal distribution. The points of each percentile curve will be obtained using the formula:


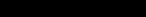

where *Zα* is the deviation from the normal equivalent for the surface of the tail *α*

#### 2.4.3 Software

All statistical analyses were performed using SPSS software version 28.0. The LMS Chart Maker Pro software version 2.3 (The Institute of Child Health, London) was used to smooth the percentile curves, according to sex and age and the medcalc software version 20.0 (Ostend, Belgium) for the constrictions of the Bland–Altman graphical method.

## 3 RESULTS

This study was conducted on 1687 Tunisian children and adolescents, where 9% refused to participate in the study and 2% suffered from chronic illnesses. Therefore, 6MWT data from 1051 children (706 boys and 795 girls) were used to obtain smoothed percentile curves by age and gender.

Tables 1 and 2 present the descriptive characteristics of the sample, which is divided into nine age groups. The percentages of boys in each age group are as follows: 9.77% (10 years), 12.46% (11 years), 10.90% (12 years), 13.17% (13 years), 11.90% (14 years), 12.04% (15 years), 10.48% (16 years), 11.33% (17 years), and 7.93% (18 years). The percentages of girls in each age group are as follows: 9.94% (10 years), 10.06% (11 years), 11.70% (12 years), 11.95% (13 years), 11.57% (14 years), 11.57% (15 years), 12.58% (16 years), 12.70% (17 years), and 7.92% (18 years).

**TABLE 1:** Characteristics of anthropometric measurements (height, body mass, BMI, and WC), PHR, RHR, and 6MWD by age group of boys participants expressed as mean and standard deviation.

Age group (years)	*n*	Height (cm)	Body mass (kg)	BMI (kg/m^2^)	WC (cm)	PHR (bpm)	RHR (bpm)	SH (cm)	LL (cm)	6MWD (m)
10	69	142.83	35.40	17.33	66.98	149.19	93.32	71.72	71.09	392.72
		±8.03	±4.39	±1.25	±5.41	±13.83	±9.66	±3.66	±8.88	±109.56
11	88	151.34***	41.45***	18.05	66.89	147.45	90.76	72.52	78.83***	415.77
		±8.16	±5.73	±1.64^b^	±5.27	±13.08	±8.09	±4.30	±8.99	±106.95
12	77	156.94***	47.47***	19.23**	67.84	151.61	89.47	74.74*	82.18	474.45
		±6.75	±5.74	±1.44	±5.21	±13.09	±8.92	±4.26	±8.03	±104.87
13	93	158.66	49.53	19.60	72.44***	144.67*	89.46	78.11***	80.59	490.60
		±7.11	±6.06^b^	±1.31^b^	±4.80	±15.26^a^	±10.76^b^	±4.57	±8.17	±125.00
14	84	164.92***	54.68***	20.10	73.41	149.52	90.27	79.04	85.78**	543.44
		±7.00	±6.12^c^	±1.53^c^	±4.62	±14.41	±10.32	±4.71^a^	±8.65	±129.53
15	85	170.32***	60.21***	20.74	75.06	146.26	89.38	83.62***	86.72	606.11*
		±9.00	±6.96	±1.65^b^	±4.80	±12.07	±11.25^b^	±4.43	±9.75	±126.62
16	74	174.88**	68.02***	22.18***	75.42	144.91	89.04	85.47	89.40	637.54
		±7.54^b^	±9.76^a^	±2.30	±4.64	±13.51	±11.25	±4.29	±8.24^a^	±122.58^a^
17	80	180.00**	74.04***	22.83	79.71***	148.16	84.20	87.61	92.36	673.55
		±7.96^c^	±8.87^c^	±1.99	±5.37	±14.73	±9.73^b^	±3.87	±9.04^c^	±128.19^c^
18	56	178.68	69.45**	21.76*	80.45	146.50	78.57*	87.62	91.00	643.41
		±7.29^a^	±9.44	±2.59	±5.52	±14.38	±9.99^c^	±3.51	±7.36^a^	±133.99

Abbreviations: 6MWD, six minutes walking distance; BMI, body mass index; LL, lengh leg; PHR, peak heart rate; RHR, resting heart rate; WC, waist circumference.

^a^ *p* < 0.01.

^b^ *p* < 0.001.

^c^ *p* < 0.0001; differences between boys and girls in the same age group.

*p < 0.01; **p < 0.001; ***p < 0.0001; differences between successive age group.

**TABLE 2:** Characteristics of anthropometric measurements (height, body mass, BMI, and WC), PHR, RHR, and 6MWD by age group of girls participants expressed as mean and standard deviation.

Age group (years)	n	Height (cm)	Body mass (kg)	BMI (kg/m^2^)	WC (cm)	PHR (bpm)	RHR (bpm)	SH (cm)	LL (cm)	6MWD (m)
10	79	145.04	37.89	17.99	66.89	148.15	96.51	70.23	74.81	440.11
		±6.29	±4.27	±1.21	±6.19	±12.22	±5.41	±3.79	±6.96	±96.16
11	80	152.09***	44.60***	19.26	67.29	153.63	94.33	73.11***	78.99	481.15
		±6.60	±5.59	±1.60	±6.51	±10.58	±6.07	±5.16	±8.67	±102.47
12	93	158.51***	50.18***	19.95	70.60*	156.99	93.41	76.18***	82.48	551.09***
		±7.45	±5.90	±1.60	±5.25	±10.59	±5.45	±4.82	±9.48	±105.19
13	95	161.54	54.65**	20.91	71.91	151.74	94.49	80.05***	81.41	605.76**
		±8.99	±7.82	±1.85	±5.47	±13.94	±5.80	±3.85	±10.73	±97.93
14	92	166.34**	60.83***	21.91	74.52	148.62	92.02	81.60	84.80	628.57
		±8.10	±8.48	±2.03	±5.37	±14.21	±6.23	±3.37	±8.79	±100.75
15	92	167.84	61.96	21.94	75.13	150.77	94.85	84.10**	83.74	621.40
		±9.62	±8.45	±1.79	±5.23	±13.19	±7.14	±4.29	±10.10	±101.83
16	100	168.65	64.01	22.41	76.42	150.60	91.14**	84.92	83.65	648.40
		±9.59	±10.18	±2.06	±6.68	±13.76	±7.49	±4.13	±10.86	±107.92
17	101	170.23	65.43	22.51	77.35	147.25	89.86	87.02*	83.18	651.09
		±7.95	±9.37	±2.04	±6.69	±15.17	±7.92	±3.95	±9.14	±94.71
18	63	172.51	68.02	22.81	80.84*	145.76	87.98	87.75	84.79	683.14
		±8.11	±9.42	±2.20	±5.77	±15.90	±8.37	±3.58	±8.56	±99.52

Abbreviations: 6MWD, six minutes walking distance; BMI, body mass index; LL, lengh leg; PHR, peak heart rate; RHR, resting heart rate; WC, waist circumference.

*p < 0.01; **p < 0.001; ***p < 0.0001; differences between successive age group.

### 3.1 Comparison between successive age groups

Significant differences (*p* < 0.05) were found in height, body mass, and SH between several consecutive age groups in both genders. For WC, differences between the 11/12 and 17/18 age groups in girls and between 12/13 and 16/17 in boys were notable. For RHR, there was a significant difference between 15/16 in girls and 17/18 in boys. Also, for the parameters BMI (11/12, 15/16, and 17/18), PHR (12/13), and LL (10/11, 13/14), the differences were noticeable only in boys. Regarding 6MWD, the differences were only observed between the age groups 11/12 and 12/13 for the girls and between 14 and 15 for the boys (Tables 1 and 2).

### 3.2 Comparison between boys and girls

Comparisons between the values of girls and boys in the same age group revealed significant differences (*p* < 0.05) in height for ages 16, 17, and 18 and in weight for ages 13, 14,16, and 17. Notable differences were found in BMI for ages 11, 13, 14, and 15 and RHR for ages 13, 15, 17, and 18. In LL, the differences were significates for age 16, 17, and 18. Parameters such as PHR and SH, also showed significant differences in only one age group, 13 and 14, respectively. With regard to 6MWD, boys in age groups 16 and 17 had significantly higher values (*p* < 0.05) than girls (Tables 1 and 2).

### 3.3 Reference values

**FIGURE 1:**
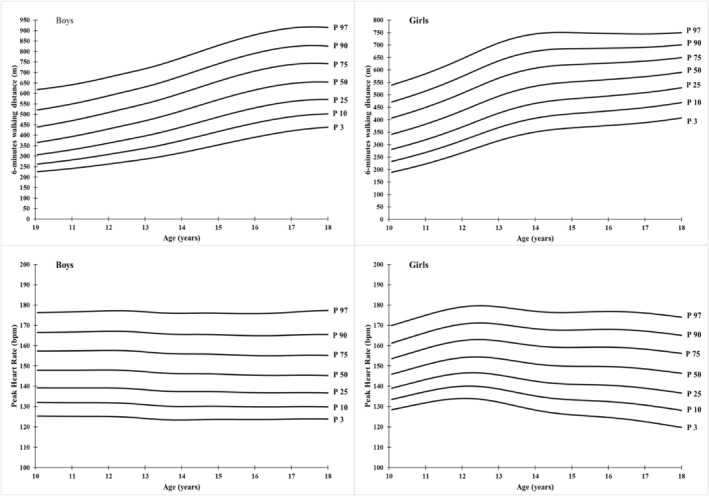
Smoothed percentiles curves of 6‐min walking distance (6MWD) and peak heart rate (PHR) for Tunisian children aged 10–18 years.

The median values of 6MWD curve range from 365,82 m (10 years) to 653,77 m (18 years) for boys and from 341,35 m (10 years) to 590,72 m (18 years) for girls. Lower percentile (P3) values range from 226,03 m (10 years) to 438,45 m (18 years) and 189,25 m (10 years) to 407,39 m (18 years) for both boys and girls, respectively. Similarly, the values of the upper percentile (P97) increased from 671,90 m (10 years) to 914,50 m (18 years) for boys and from 539,75 m (10 years) to 750,20 m (18 years) for girls (Table 3 and Figure 1).

**TABLE 3:** L, M, and S values and percentile curves of 6MWD (m) for Tunisian children aged 10–18 years.

Age (years)	L	M	S	P3	P10	P25	P50	P75	P90	P97
Boys										
10	−0.17	365.82	0.27	226.03	262.40	306.41	365.82	439.14	520.20	617.90
11	0.01	394.26	0.26	241.81	282.64	330.95	394.26	469.54	549.35	641.29
12	0.18	430.43	0.25	262.74	308.97	362.41	430.43	508.60	588.55	677.31
13	0.33	469.44	0.24	286.42	338.25	396.82	469.44	550.48	630.98	717.83
14	0.46	517.06	0.23	317.09	375.13	439.31	517.06	601.65	683.70	770.26
15	0.56	569.82	0.22	354.04	417.94	487.34	569.82	657.87	741.79	828.89
16	0.62	616.43	0.21	390.60	458.27	530.96	616.43	706.69	791.89	879.59
17	0.59	647.16	0.20	420.99	488.68	561.45	647.16	737.84	823.63	912.10
18	0.48	653.77	0.19	438.45	502.18	571.37	653.77	742.01	826.45	914.50
Girls										
10	0.48	341.35	0.27	189.25	232.73	281.52	341.35	407.13	471.47	539.75
11	0.50	382.39	0.25	222.79	268.96	320.19	382.39	450.14	515.89	585.21
12	0.51	436.35	0.23	267.46	316.89	371.15	436.35	506.70	574.41	645.33
13	0.55	494.91	0.21	315.91	369.06	426.63	494.91	567.68	636.94	708.78
14	0.65	535.11	0.20	350.82	406.56	465.91	535.11	607.62	675.60	745.15
15	0.80	552.05	0.18	367.31	424.50	484.08	552.05	621.77	685.92	750.45
16	1.00	561.84	0.17	376.92	435.85	495.53	561.84	628.14	687.80	746.68
17	1.24	573.32	0.16	388.52	449.29	508.93	573.32	636.04	691.26	744.77
18	1.47	590.72	0.15	407.39	469.39	528.49	590.72	650.00	701.27	750.20

Abbreviations: 6MWD, six minutes walking distance; L, Lambda; M, Median; P, Percentile; S, sigma‐coefficient of variation.

The 50th percentile values for PHR decrease in boys from 147.90 bpm at age 10 to 145.25 bpm at age 18 and increase in girls from 145.90 bpm at age 10 to 146.36 bpm at age 18. The 3rd percentile values range from 125.40 bpm at age 10 years to 123.91 bpm at age 18 years in boys and from 128.45 bpm at age 10 years to 119.81 bpm at age 18 years in girls. In addition, 97th percentile values increased from 176.25 bpm at age 10 years to 177.39 bpm at age 18 years for boys and from 169.86 bpm at age 10 years to 174.00 bpm at age 18 years for girls (Table 4 and Figure 1).

**TABLE 4:** L, M, and S values and percentile curves of PHR (bpm) for Tunisian children aged 10–18 years.

Age (years)	L	M	S	P3	P10	P25	P50	P75	P90	P97
Boys										
10	−0.36	147.90	0.09	125.40	132.03	139.24	147.90	157.30	166.47	176.25
11	−0.38	147.91	0.09	125.27	131.93	139.19	147.91	157.41	166.70	176.62
12	−0.40	148.01	0.09	125.17	131.87	139.19	148.01	157.63	167.06	177.16
13	−0.43	147.16	0.09	124.23	130.94	138.29	147.16	156.87	166.41	176.66
14	−0.51	146.18	0.09	123.46	130.08	137.36	146.18	155.90	165.50	175.88
15	−0.69	146.00	0.09	123.75	130.18	137.30	146.00	155.67	165.35	175.93
16	−0.89	145.42	0.09	123.69	129.91	136.85	145.42	155.06	164.82	175.67
17	−1.09	145.39	0.09	123.90	129.99	136.84	145.39	155.13	165.14	176.45
18	−1.28	145.25	0.09	123.91	129.90	136.68	145.25	155.15	165.50	177.39
Girls										
10	−1.27	145.90	0.07	128.45	133.48	139.05	145.90	153.57	161.30	169.86
11	−0.88	150.57	0.07	131.87	137.34	143.32	150.57	158.54	166.42	174.95
12	−0.47	154.15	0.08	134.00	139.98	146.44	154.15	162.46	170.53	179.08
13	−0.12	153.65	0.08	132.17	138.63	145.53	153.65	162.29	170.53	179.12
14	0.17	151.00	0.09	128.33	135.22	142.51	151.00	159.90	168.28	176.91
15	0.38	149.87	0.09	125.99	133.32	141.01	149.87	159.06	167.63	176.36
16	0.54	149.71	0.09	124.70	132.44	140.50	149.71	159.20	167.96	176.83
17	0.67	148.55	0.10	122.64	130.72	139.07	148.55	158.23	167.11	176.04
18	0.78	146.36	0.10	119.81	128.15	136.71	146.36	156.15	165.08	174.00

Abbreviations: L, Lambda; M, Median, P, Percentile; PHR, peak heart rate; S, sigma‐coefficient of variation.

**FIGURE 2:**
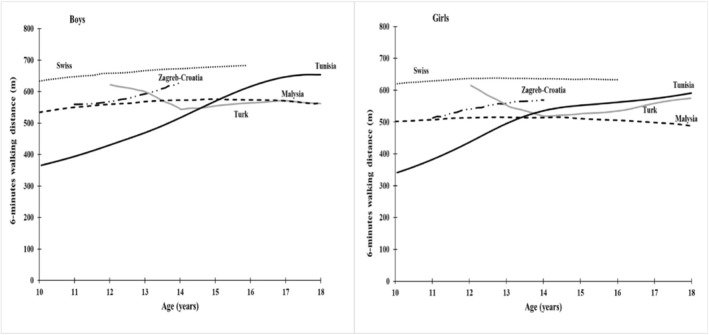
Comparison of median (50th percentile) 6‐min walking distance (6MWD) curves of Tunisian children with these of Swiss, Zagreb‐Croatia, Turk, and Malaysia.

By comparing the 50th percentile of our reference with that of other countries, the maximum differences with the Swiss reference reached 278.2 m for girls and 267.43 m for boys at 10 years of age (Figure 2).

### 3.4 6MWD prediction equations

**FIGURE 3:**
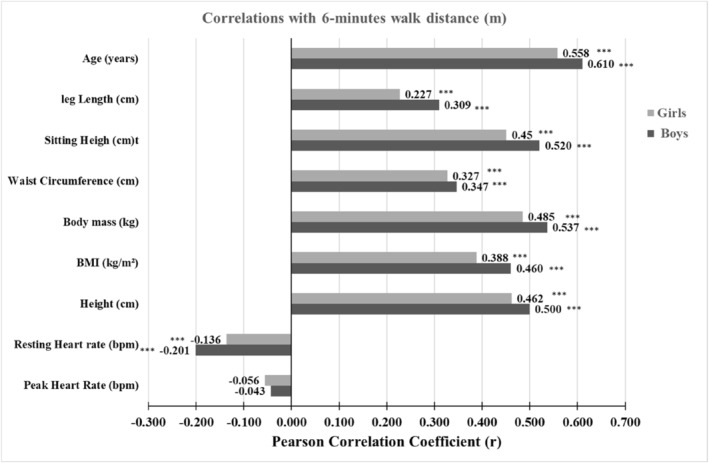
Pearson correlation coefficients between Observed 6 minutes walking distance (6MWD) (m) and the independents variables.

Pearson correlation coefficients between 6MWD and several variables (age, weight, body mass, BMI, WC, PHR, RHR, SH, and LL) are shown in Figure 3. All variables showed a strong positive correlation (*p* < 0.001) with 6MWD (*r* ranged from 0.227 to 0.558 for girls and from 0.309 to 0.610 for boys), except RHR, which showed a strong negative correlation (girls: *r* = − 0.136; boys: *r* = − 0.201; *p* < 0.001), while PHR showed a weak correlation (*p* > 0.05).

Stepwise regression analysis examined predictors of 6MWD and yielded four different models, two of which applied to girls and two to boys. In girls, model 1 had an *R* ^2^ of 0.312 and an Standard Error of Estimate (SEE) of 103.66. Similarly, model 2 had an *R* ^2^ of 0.325 and an SEE of 102.70. In boys, model 1 had an *R* ^2^ of 0.372 and an SEE of 122.13, whereas model 2 had an *R* ^2^ of 0.378 and an SEE of 121.60 (Table 5). Based on the results, which indicated similar values for *R* ^2^ and SEE, we decided to select model 1 for both girls and boys. This decision was made because model 1 relies on only one variable, that is, age.

**TABLE 5:** Stepwise multiple linear regression model for 6MWD.

Models		Unstandardized coefficients		Standardized coefficients	*t*	Significance	*R*	*R* ^2^	Adjusted *R* ^2^	Standard error of estimate
		*B*	Standard error	*β*						
Boys										
1^a^	(Constante)	66.181	27.193		2.434	0.015	0.610	0.372	0.371	122.13
	AGE	38.142	1.868	0.610	20.417	0.000				
2	(Constante)	177.647	49.481		3.590	0.000	0.615	0.378	0.377	121.60
	AGE	42.613	2.494	0.681	17.088	0.000				
	WC	−2.408	0.895	−0.107	−2.691	0.007				
Girls										
1^a^	(Constante)	105.535	22.031		4.790	0.000	0.558	0.312	0.311	103.66
	AGE	28.390	1.499	0.558	18.945	0.000				
2	(Constante)	−91.390	53.950		−1.694	0.091	0.570	0.325	0.323	102.70
	AGE	22.997	2.007	0.452	11.456	0.000				
	Height	1.690	0.424	0.158	3.991	0.000				

*Note*: Both models had almost similar *R* ^2^ and SEE values for each gender, we chose the simpler model (a) that included only age as a predictor of 6MWD.

Abbreviations: 6MWD, six minutes walking distance; SEE, Standard Error of Estimate.

**FIGURE 4:**
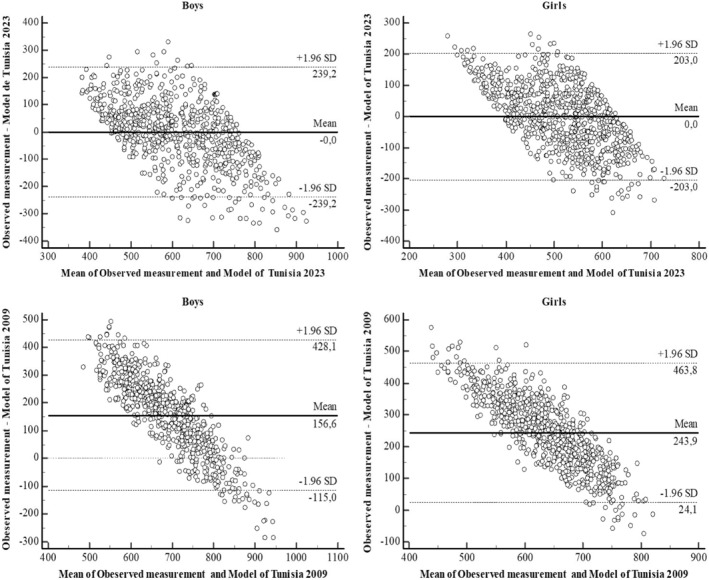
Bland–Altman plots of the difference between observed and predicted values using two Models: Model of Tunisia 2023 and model of Tunisia 2009.

Figure 4 illustrated the degree of agreement between model 1 and measured 6MWD values for both genders. For boys, model 1 showed a discrepancy of −0.002 m, with an upper limit of agreement of +239.2 m and a lower limit of agreement of −239.2 m. Conversely, for girls, model 1 showed a deviation of −0.003 m, with an upper limit of agreement of +203 m and a lower limit of agreement of −203 m. In addition, Figure 4 displayed the degree of agreement between the Tunisian 2009 model and the measured values of 6MWD, which showed a deviation of +156.6 m, with an upper limit of agreement of +428.1 m and a lower limit of agreement of −115.1 m in boys, while in girls the difference is equal to 243.9 and the upper and lower limits are 463.8 and −24.1 m, respectively.

## 4 DISCUSSIONS

In this study, we have established normative values for the Six‐Minute Walk Test (6MWT) among Tunisian children and adolescents, a significant step in understanding the functional capacity in each age group within the gender. Our aim of this study was to establish reference curves of 6MWD and PHR and to formulate a prediction equation for children and adolescents.

The 3rd, 50th, and 97th percentile values of 6MWD in boys showed a continuous increase with age, through a difference of approximately 24.13 ± 10.61 m, 31.18 ± 19.85 m, and 31 0.35 ± 24.16 m per year, respectively, resulting in an overall rate of increase +96.09%, +76.72%, and +45.66% between 10 and 18 years. In girls, the variations are about 25.63 ± 14.95 m, 28.88 ± 19.57 m, and 23.85 ± 27.40 m per year, respectively, resulting in an overall rate of increase of +121.87%, +76.15%, and +39.76%. The increase in 6MWD with age in adolescents can be attributed to several factors. (1) Musculoskeletal development, including increased muscle mass, strength, and muscular endurance. These changes allow a better ability to carry out prolonged physical effort (Caselli et al., 2021; de Lima et al., 2022; Philippou et al., 2007). (2) Adolescents also develop their energy system, including the efficient use of energy sources, such as carbohydrates and fats, to produce energy during exercise (DeBoer, 2019; Desbrow, 2021; Troiano et al., 2000). (3) Cardiorespiratory capacity also develops with adolescence age. Increased lung capacity (Peralta et al., 2019; Wang et al., 1993), better tissue oxygenation, and adaptation of the heart (Maresh, 1948; Viru et al., 1999) to higher exercise loads contribute to better cardiorespiratory endurance. Despite these adaptations, the variations of the PHR percentile values of the walking test with age are minimal. Indeed, the values of the 3rd, 50th, and 97th percentiles in percentiles vary with rates respectively of −1.45%, −2.11%, and +0.85% for boys and −8.00%, ‐ 0.60%, and +1.67% for girls. We also found significant differences in 6MWD by sex at ages 16 and 17. Boys had a longer average distance than girls. This can be attributed to greater muscle mass due to sex hormones (Ramos et al., 1998), such as testosterone which can influence muscle strength and physical performance, and greater height which is more commonly found in boys (Kanburoglu et al., 2014; Li et al., 2007; Wardhani et al., 2023). Notably LL is a primary determinant of stride length (Enright & Sherrill, 1998; Wardhani et al., 2023). This is confirmed in our study where we found significant differences of raw values between boys and girls at ages 16 (*p* < 0.01) and 17 (*p* < 0.001). This is also corresponding to the maximum deviations of 73.84 and 63.05 m, respectively between the smoothed 50th percentiles.

The large differences found between the 50th percentiles of our references and those of other countries (Switzerland, Croatia, Türkiye, and Malaysia) especially at the age of 10 years may be due to various factors. (1) Genetic factors that can influence the physical condition and physical performance of children (Guth & Roth, 2013; Semenova et al., 2023). (2) Socio‐cultural and socio‐economic factor (Larrinaga‐Undabarrena et al., 2023; Lindquist et al., 1999; Pascual et al., 2009) influence the level of physical activity healthy children and adolescent through access to sports facilities (Giles‐Corti & Donovan, 2002) and the choice of healthy diets (Cvetković et al., 2021). Higher levels of physical activity may translate into better performance on the six‐minute test. (3) Sample size (Cole, 2021): If the number of subjects in each age group is large, the smoothed percentiles more accurately describe the trend of the measurements. (4) Technique for smoothing curves: In our study, we used the LMS method (Cole & Green, 1992; Ghouili et al., 2021) to develop smoothed percentiles, whereas in Turkish reference the smoothing technique was established by a curvilinear regression model (Kanburoglu et al., 2014).

Several variables, including anthropometric parameters (weight, height, BMI, WC, SH, and LL), age, and RHR, were examined to identify the best predictor of 6MWD. Our analysis revealed that age is the most significant predictor in girls and boys, with correlations of 0.56 (*p* < 0.001) and 0.61 (*p* < 0.001), respectively. Previous studies have found varying degrees of correlation between 6MWD and across different age groups of children. For instance, a study conducted on Turkish children aged 6–12 years showed a strong correlation coefficient of 0.76 (*p* < 0.001) (Özcan Kahraman et al., 2019). Similarly, for the same age group, another study on Brazilian children and adolescents from Porto Alegre reported a correlation coefficient of 0.51 (*p* < 0.001) (Priesnitz et al., 2009). However, in the case of Croatian children located in the city of Zagreb (aged 11–14 years) a weaker correlation coefficient of 0.24 (*p* < 0.001) was observed (Kasović et al., 2021). In addition, a study conducted on a wider age range of 6–75 years reported an even more modest correlation of 0.25 (*p* < 0.001) (Agarwal et al., 2023).

In our study, we developed four models to predict 6MWD. For girls, we developed two models: one based only on age, with an *R* ^2^ of 0.312 and an SEE of 103.66, and the other including both age and height as independent variables, with an *R* ^2^ of 0.325 and an SEE of 102.70. For boys, the first model that included only age as a predictor achieved an *R* ^2^ of 0.372 with an SEE of 122.13, and the second model that included both age and WC had an *R* ^2^ of 0.378 and an SEE of 121.60. Since both models had similar *R* ^2^ and SEE values, we decided to use the simpler model that only included age as a predictor of 6MWD. Related research has examined different models with varying degrees of predictive accuracy. A British study found that age alone accounted for 41% of the variation in 6MWD, and with the inclusion of weight and height, this rate increased to 44% (Lammers et al., 2008). A more recent study, which included a wide age range from 6 to 75 years, found that a model that included age and sex explained 47% of the variability in 6MWD (*R* ^2^ = 0.475). Including height in the equation did not significantly improve predictive power (*R* ^2^ = 0.478) (Agarwal et al., 2023). In addition, a study conducted on 161 healthy Brazilian children aged 6–13 years from local elementary and secondary schools developed models that based on age, weight, and height. In boys, the model explained 49% of the variability in 6MWD, with an SEE of 56.55, while in girls the variability was 33%, with an SEE of 56.47 (Oliveira et al., 2013). Another study focused on Caucasian children revealed age, weight, and height to be significant predictors (Ulrich et al., 2013).

The last study conducted in 2009 on healthy Tunisian children aged 6–16 years. Several anthropometric and spirometric parameters were measured. A simplified equation with three common parameters: age, height, and weight were established for girls, boys, and the total sample. This model appears to explain 60% of the 6MWD variability (Ben Saad, Prefaut, Missaoui, et al., 2009a).

When comparing the predicted values to the observed 6MWD values, our analysis shows notable differences between the models in our study and those developed in 2009. In particular, our models show a minimal bias tending toward zero (boys: −0.002 ± 122.05; girls: −0.003 ± 103.59) between the predicted values and the observed values. In contrast, the equation formulated in 2009 systematically overestimates distances, with substantial differences of 165.56 ± 135.55 for boys and 243.94 ± 112.19 for girls. In addition, it is important to note that the concordance limits with the 2009 model are considerably wider compared to our model, and this is true for both boys and girls' subjects. The difference in reliability between the two models can be attributed mainly to the difference in sample selection. In the model developed in 2009, they included a sample of 200 children, with 100 subjects split for each gender and covering a wide age range from 6 to 16 years old (Ben Saad, Prefaut, Missaoui, et al., 2009a). This model raises questions about its applicability to a broader population of Tunisian children, particularly with regard to the 16–18‐year age group (Serdar et al., 2021). Therefore, the reference equation that was established in 2009 cannot be generalized to the entire North African region, not even to the entire Tunisia (Krejcie & Morgan, 1970).

### 4.1 Limitations

The study provides valuable insights into the cardiopulmonary health capacities of Tunisian children and adolescents, yet there are key limitations that merit attention for a comprehensive understanding of the results.

Firstly, the regional focus of the study, encompassing only Tunisia, presents a limitation in terms of full demographic representation of similar Middle East and North African (MENA) countries. While this selection includes diverse areas, it may not completely capture the entire spectrum of the MENA region pediatric population. Factors such as regional climate variations, degrees of urbanization, and differential access to healthcare and sports facilities can influence physical performance measures such as those assessed in the 6MWT. As a result, the study's findings, although significant, might not entirely reflect the physical performance nuances of all MENA regions.

Secondly, the study's methodology, while meticulously controlling for key variables such as age, height, and body mass, did not extensively explore other influential factors that can impact physical performance and cardiovascular health. Aspects such as nutritional status, socioeconomic background, and lifestyle habits outside of school, including varying levels of physical activity, play crucial roles in shaping children's physical capabilities. The absence of these variables in the study's framework suggests a potential avenue for future research. Investigating these factors in more depth could provide a more rounded view of the determinants of physical performance among children and adolescents, offering critical insights for targeted interventions and policy‐making in pediatric health and physical education.

## 5 CONCLUSION

This study establishes vital normative values and predictive models for the 6MWT and PHR in Tunisian children and adolescents. The developed percentile curves and age‐based prediction equations offer practical tools for clinicians and researchers, enabling precise assessment and monitoring of cardiovascular functional capacity. These tools may facilitate timely interventions and may fill a crucial gap in region‐specific data. Moreover, the findings support effective health strategies by allowing meaningful comparisons with international standards, ultimately contributing to improved health outcomes for the youth.

## AUTHOR CONTRIBUTIONS

**Hatem Ghouili** contributed to the conceptualization and design of the study, interpretation of the data, and critical drafting and revising of the manuscript; **Mohamed Ben Aissa**, **Noomen Guelmami** and **Ismail Dergaa** conducted the statistical analyses and contributed to the interpretation of the data and critical revision of the manuscript; **Amel Dridi**, **Zouhaier Farhani**, **Nejmeddine Ouerghi**, **Nadhir Hammami**, **Anissa Bouassida**, **Katja Weiss**, **Thomas Rosemann**, **Nizar Souissi**, **Lamia Ben Ezzeddine** and **Beat Knechtle** contributed to the interpretation of the data and critical revision of the manuscript. All authors have read and approved the final version of the manuscript, and agree with the order of presentation of the authors.

## CONFLICT OF INTEREST STATEMENT

No conflicts of interest, financial or otherwise, are declared by the authors.

## CONSENT TO PARTICIPATE

Prior to the commencement of the study, informed consent was obtained from all participants' parents or legal guardians. This process involved a comprehensive explanation of the study's aims, procedures, potential benefits, and any associated risks. Parents or guardians were assured that participation in the study was entirely voluntary and that they had the right to withdraw their child from the study at any point without any adverse consequences. Each parent or guardian provided written consent, acknowledging their understanding and agreement for their child's participation in the study. This consent procedure was conducted in accordance with the ethical guidelines set forth by the Higher Institute of Sport and Physical Education of Kef, ensuring adherence to the international standards for research involving minors.

## Data Availability

The data that support the findings of this study are openly available upon request from the corresponding author.
